# Supplementary material for: A terahertz-driven non-equilibrium phase transition in a room temperature atomic vapour
Source: Nat Commun. 2018 Sep 3;9:3567. doi: 10.1038/s41467-018-05597-4 (PMC6120943; doi:10.1038/s41467-018-05597-4)
Supplement: Supplementary file 1 — Supplementary Information [file 41467_2018_5597_MOESM1_ESM.pdf]

A terahertz-driven non-equilibrium phase transition in a room temperature atomic vapour

Supplementary information

Wade *et al.*

I. SUPPLEMENTARY NOTE 1: DERIVATION OF THE PHENOMENOLOGICAL EQUATIONS

We consider a minimal model consisting of three levels depicted in Fig. 2d of the main text. As stated there, these three levels correspond to the ground state $|0\rangle$, the first Rydberg state $|\mathrm{R}\rangle$ and the second one $|\mathrm{T}\rangle$. A complete set of operators in this basis is given by the $\sigma_{ab,k} = |a\rangle_k\, {}_k\langle b|$ with $a, b \in \{0, \mathrm{R}, \mathrm{T}\}$ and k is an index labelling the k-th atom. This choice is convenient for calculations, since it allows to exploit the orthogonality condition

$$\sigma_{ab,k}\sigma_{cd,k} = \delta_{bc}\sigma_{ad,k}, \tag{1}$$

where δ_{bc} is the Kronecker symbol. For later convenience, we also introduce the more physical basis of operators

$$\begin{aligned}
\hat{n}_{\mathrm{R},k} &= |\mathrm{R}\rangle_k\, {}_k\langle \mathrm{R}|, \\
\hat{n}_{\mathrm{T},k} &= |\mathrm{T}\rangle_k\, {}_k\langle \mathrm{T}|, \\
\hat{\sigma}^x_{\mathrm{R},k} &= |0\rangle_k\, {}_k\langle \mathrm{R}| + |\mathrm{R}\rangle_k\, {}_k\langle 0|, \\
\hat{\sigma}^y_{\mathrm{R},k} &= i\,|0\rangle_k\, {}_k\langle \mathrm{R}| - i\,|\mathrm{R}\rangle_k\, {}_k\langle 0|, \\
\hat{\sigma}^x_{\mathrm{RT},k} &= |\mathrm{R}\rangle_k\, {}_k\langle \mathrm{T}| + |\mathrm{T}\rangle_k\, {}_k\langle \mathrm{R}|, \\
\hat{\sigma}^y_{\mathrm{RT},k} &= i\,|\mathrm{R}\rangle_k\, {}_k\langle \mathrm{T}| - i\,|\mathrm{T}\rangle_k\, {}_k\langle \mathrm{R}|, \\
\hat{\sigma}^x_{\mathrm{T},k} &= |0\rangle_k\, {}_k\langle \mathrm{T}| + |\mathrm{T}\rangle_k\, {}_k\langle 0|, \\
\hat{\sigma}^y_{\mathrm{T},k} &= i\,|0\rangle_k\, {}_k\langle \mathrm{T}| - i\,|\mathrm{T}\rangle_k\, {}_k\langle 0|,
\end{aligned} \tag{2}$$

consisting of the two populations of the Rydberg states and six coherences between pairs of levels.

We write an effective Hamiltonian $\hat{H}_{TOT} = \hat{H} + \hat{H}_{\mathrm{int}}$, where $\hat{H}$ describes the light-atoms coupling, whereas interatomic interactions are encoded in $\hat{H}_{\mathrm{int}}$. In the rotating wave approximation, where terms counter-rotating with respect to the incident radiation are discarded, the former takes the form

$$\begin{aligned}
\hat{H} &= \sum_k \left[\left[\frac{\Omega_\mathrm{R}}{2}\sigma_{0\mathrm{R},k} + \frac{\Omega_\mathrm{T}}{2}\sigma_{\mathrm{RT},k} + h.c. \right] - D_\mathrm{R}\sigma_{\mathrm{RR},k} - D_{\mathrm{RT}}\sigma_{\mathrm{TT},k} \right] = \\
&= \sum_k \left[\frac{\Omega_\mathrm{R}}{2}\hat{\sigma}^x_{\mathrm{R},k} + \frac{\Omega_\mathrm{T}}{2}\hat{\sigma}^x_{\mathrm{T},k} - D_\mathrm{R}\hat{n}_{\mathrm{R},k} - D_{\mathrm{RT}}\hat{n}_{\mathrm{T},k} \right],
\end{aligned} \tag{3}$$

where Ω_R (Ω_T) is the Rabi frequency of the coupling between levels $|0\rangle$ and $|\mathrm{R}\rangle$ ($|\mathrm{T}\rangle$), D_R is the detuning of the laser with respect to the former transition, D_T the one with respect to the latter, and $D_{\mathrm{RT}} = D_\mathrm{R} - D_\mathrm{T}$. Of course, in our simple model Ω_R

and D_{R} account for the combined Rabi frequency and detuning of the more complex three-laser scheme actually employed in the experiment to excite atoms from their electronic ground state to the selected Rydberg level. In the interaction part we include density-density potentials of the van-der-Waals type on the Rydberg levels, so that

$$\hat{H}_{\mathrm{int}} = \frac{1}{2}\sum_{k\neq p}\left[\frac{C_6^{\mathrm{R}}}{|\vec{r}_k-\vec{r}_p|^6}\hat{n}_{\mathrm{R},k}\hat{n}_{\mathrm{R},p} + \frac{C_6^{\mathrm{T}}}{|\vec{r}_k-\vec{r}_p|^6}\hat{n}_{\mathrm{T},k}\hat{n}_{\mathrm{T},p}\right], \tag{4}$$

where $\vec{r}_k$ denotes the position of the k-th atom and $C_6^{\mathrm{R(T)}}$ encodes the strength of the interactions between atoms in level $|\mathrm{R}\rangle$ ($|\mathrm{T}\rangle$).

Additionally, we consider dissipative processes in the form of radiative decay from the Rydberg levels to the ground state. We further assume them to be Markovian and spatially uncorrelated, so that we can describe them via jump operators of the form

$$L_{\mathrm{R},k} = \sqrt{\Gamma_{\mathrm{R}}}\,\sigma_{0\mathrm{R},k} \quad \text{and} \quad L_{\mathrm{T},k} = \sqrt{\Gamma_{\mathrm{T}}}\,\sigma_{0\mathrm{T},k}, \tag{5}$$

with Γ_{R}, Γ_{T} being the respective rates. The evolution of any given operator $\mathcal{O}$ can then be described via an adjoint Lindblad equation

$$\dot{\mathcal{O}} = i\left[\hat{H}_{TOT},\mathcal{O}\right] + \sum_{\alpha=\mathrm{R,T}}\sum_{k}\left[L^{\dagger}_{\alpha,k}\mathcal{O}L_{\alpha,k} - \frac{1}{2}\left\{L^{\dagger}_{\alpha,k}L_{\alpha,k},\mathcal{O}\right\}\right], \tag{6}$$

where $[A,B] = AB - BA$ denotes commutation and $\{A,B\} = AB + BA$ anticommutation. The calculation is more easily performed in the σ_{ab} basis, therefrom the equations of motion for the observables (supplementary Eqs. (2)) can be derived tak-

ing the appropriate linear combinations. The result is

$$\dot{\hat{n}}_{\mathrm{R},k} = \Omega_\mathrm{R}\hat{\sigma}^y_{\mathrm{R},k} - \Omega_\mathrm{T}\hat{\sigma}^y_{\mathrm{RT},k} - \Gamma_\mathrm{R}\hat{n}_{\mathrm{R},k} \tag{7}$$

$$\dot{\hat{n}}_{\mathrm{T},k} = \Omega_\mathrm{T}\hat{\sigma}^y_{\mathrm{RT},k} - \Gamma_\mathrm{T}\hat{n}_{\mathrm{T},k} \tag{8}$$

$$\dot{\hat{\sigma}}^x_{\mathrm{R},k} = D_\mathrm{R}\hat{\sigma}^y_{\mathrm{R},k} - \Omega_\mathrm{T}\hat{\sigma}^y_{\mathrm{T},k} - \sum_{p\neq k}\frac{C_6^\mathrm{R}}{|\vec{r}_k-\vec{r}_p|^6}\hat{\sigma}^y_{\mathrm{R},k}\hat{n}_{\mathrm{R},p} - \frac{\Gamma_\mathrm{R}}{2}\hat{\sigma}^x_{\mathrm{R},k} \tag{9}$$

$$\dot{\hat{\sigma}}^y_{\mathrm{R},k} = -D_\mathrm{R}\hat{\sigma}^x_{\mathrm{R},k} + \Omega_\mathrm{T}\hat{\sigma}^x_{\mathrm{T},k} - 2\Omega_\mathrm{R}(2\hat{n}_{\mathrm{R},k} + \hat{n}_{\mathrm{T},k} - 1) + \sum_{p\neq k}\frac{C_6^\mathrm{R}}{|\vec{r}_k-\vec{r}_p|^6}\hat{\sigma}^x_{\mathrm{R},k}\hat{n}_{\mathrm{R},p} + \\ - \frac{\Gamma_\mathrm{R}}{2}\hat{\sigma}^y_{\mathrm{R},k} \tag{10}$$

$$\dot{\hat{\sigma}}^x_{\mathrm{RT},k} = -D_\mathrm{T}\hat{\sigma}^y_{\mathrm{RT},k} + \Omega_\mathrm{R}\hat{\sigma}^y_{\mathrm{T},k} + \hat{\sigma}^y_{\mathrm{RT},k}\sum_{p\neq k}\left(\frac{C_6^\mathrm{R}}{|\vec{r}_k-\vec{r}_p|^6}\hat{n}_{\mathrm{R},p} - \frac{C_6^\mathrm{T}}{|\vec{r}_k-\vec{r}_p|^6}\hat{n}_{\mathrm{T},p}\right) + \\ - \frac{\Gamma_\mathrm{R}+\Gamma_\mathrm{T}}{2}\hat{\sigma}^x_{\mathrm{RT},k} \tag{11}$$

$$\dot{\hat{\sigma}}^y_{\mathrm{RT},k} = D_\mathrm{T}\hat{\sigma}^x_{\mathrm{RT},k} - \Omega_\mathrm{R}\hat{\sigma}^x_{\mathrm{T},k} - 2\Omega_\mathrm{T}(\hat{n}_{\mathrm{T},k} - \hat{n}_{\mathrm{R},k}) + \\ - \hat{\sigma}^x_{\mathrm{RT},k}\sum_{p\neq k}\left(\frac{C_6^\mathrm{R}}{|\vec{r}_k-\vec{r}_p|^6}\hat{n}_{\mathrm{R},p} - \frac{C_6^\mathrm{T}}{|\vec{r}_k-\vec{r}_p|^6}\hat{n}_{\mathrm{T},p}\right) - \frac{\Gamma_\mathrm{R}+\Gamma_\mathrm{T}}{2}\hat{\sigma}^y_{\mathrm{RT},k} \tag{12}$$

$$\dot{\hat{\sigma}}^x_{\mathrm{T},k} = D_\mathrm{RT}\hat{\sigma}^y_{\mathrm{T},k} + \Omega_\mathrm{R}\hat{\sigma}^y_{\mathrm{RT},k} - \Omega_\mathrm{T}\hat{\sigma}^y_{\mathrm{R},k} - \sum_{p\neq k}\frac{C_6^\mathrm{R}}{|\vec{r}_k-\vec{r}_p|^6}\hat{\sigma}^y_{\mathrm{T},k}\hat{n}_{\mathrm{T},p} - \frac{\Gamma_\mathrm{T}}{2}\hat{\sigma}^x_{\mathrm{T},k} \tag{13}$$

$$\dot{\hat{\sigma}}^y_{\mathrm{T},k} = -D_\mathrm{RT}\hat{\sigma}^x_{\mathrm{T},k} - \Omega_\mathrm{R}\hat{\sigma}^x_{\mathrm{RT},k} + \Omega_\mathrm{T}\hat{\sigma}^x_{\mathrm{R},k} + \sum_{p\neq k}\frac{C_6^\mathrm{R}}{|\vec{r}_k-\vec{r}_p|^6}\hat{\sigma}^x_{\mathrm{T},k}\hat{n}_{\mathrm{T},p} - \frac{\Gamma_\mathrm{T}}{2}\hat{\sigma}^y_{\mathrm{T},k}. \tag{14}$$

At this level, these equations are still exact. We now perform a mean-field decoupling, i.e., we take the expectation values of these observables and neglect correlations between different sites. Furthermore, we assume the system is (to a good approximation) translationally invariant in the bulk and we restrict to uniform averages. This means that we replace correlations $\left\langle \hat{\mathcal{O}}_k \hat{\mathcal{O}}'_p \right\rangle$ with $\left\langle \hat{\mathcal{O}}_k \right\rangle \left\langle \hat{\mathcal{O}}'_p \right\rangle$ and we set $\left\langle \hat{\mathcal{O}}_k \right\rangle = \mathcal{O}$ $\forall k$. Introducing the shorthand notation

$$V_{\mathrm{R(T)}} = \sum_{p\neq k}\frac{C_6^{\mathrm{R(T)}}}{|\vec{r}_k-\vec{r}_p|^6}, \tag{15}$$

the equations above are reduced to a closed set of eight non-linear coupled equations:

$$\dot{n}_{\rm R} = \Omega_{\rm R}\sigma^y_{\rm R} - \Omega_{\rm T}\sigma^y_{\rm RT} - \Gamma_{\rm R} n_{\rm R} \tag{16}$$

$$\dot{n}_{\rm T} = \Omega_{\rm T}\sigma^y_{\rm RT} - \Gamma_{\rm T} n_{\rm T} \tag{17}$$

$$\dot{\sigma}^x_{\rm R} = (D_{\rm R} - V_{\rm R} n_{\rm R})\,\sigma^y_{\rm R} - \Omega_{\rm T}\sigma^y_{\rm T} - \frac{\Gamma_{\rm R}}{2}\sigma^x_{\rm R} \tag{18}$$

$$\dot{\sigma}^y_{\rm R} = -\,(D_{\rm R} - V_{\rm R} n_{\rm R})\,\sigma^x_{\rm R} + \Omega_{\rm T}\sigma^x_{\rm T} - 2\Omega_{\rm R}(2n_{\rm R} + n_{\rm T} - 1) - \frac{\Gamma_{\rm R}}{2}\sigma^y_{\rm R} \tag{19}$$

$$\dot{\sigma}^x_{\rm RT} = -\,(D_{\rm T} - V_{\rm R} n_{\rm R} + V_{\rm T} n_{\rm T})\,\sigma^y_{\rm RT} + \Omega_{\rm R}\sigma^y_{\rm T} - \frac{\Gamma_{\rm R} + \Gamma_{\rm T}}{2}\sigma^x_{\rm RT} \tag{20}$$

$$\dot{\sigma}^y_{\rm RT} = (D_{\rm T} - V_{\rm R} n_{\rm R} + V_{\rm T} n_{\rm T})\,\sigma^x_{\rm RT} - \Omega_{\rm R}\sigma^x_{\rm T} - 2\Omega_{\rm T}(n_{\rm T} - n_{\rm R}) - \frac{\Gamma_{\rm R} + \Gamma_{\rm T}}{2}\sigma^y_{\rm RT} \tag{21}$$

$$\dot{\sigma}^x_{\rm T} = (D_{\rm RT} - V_{\rm T} n_{\rm T})\,\sigma^y_{\rm T} + \Omega_{\rm R}\sigma^y_{\rm RT} - \Omega_{\rm T}\sigma^y_{\rm R} - \frac{\Gamma_{\rm T}}{2}\sigma^x_{\rm T} \tag{22}$$

$$\dot{\sigma}^y_{\rm T} = -\,(D_{\rm RT} - V_{\rm T} n_{\rm T})\,\sigma^x_{\rm T} - \Omega_{\rm R}\sigma^x_{\rm RT} + \Omega_{\rm T}\sigma^x_{\rm R} - \frac{\Gamma_{\rm T}}{2}\sigma^y_{\rm T}. \tag{23}$$

In the mean-field equations we have collected terms proportional to the same variables in order to highlight the fact that, in this picture, the interactions merely act by shifting the energy levels or, equivalently, by rescaling the detunings according to $D_{\rm R} \to D_{\rm R} - V_{\rm R} n_{\rm R}$ and $D_{\rm RT} \to D_{\rm RT} - V_{\rm T} n_{\rm T}$. Note that, since $D_{\rm RT} = D_{\rm R} - D_{\rm T}$, this automatically implies $D_{\rm T} \to D_{\rm T} - V_{\rm R} n_{\rm R} + V_{\rm T} n_{\rm T}$.

We account for ionization processes in a similar, simplified way by assuming that portions $q_{\rm R}$ and $q_{\rm T}$ of the atoms in levels $|{\rm R}\rangle$ and $|{\rm T}\rangle$ get ionized by interatomic collisions. According to this prescription, the correct normalization for the densities is now

$$\underbrace{n_0}_{\text{ground state atom density}} + \underbrace{n_{\rm R} + n_{\rm T}}_{\text{Rydberg atom density}} + \underbrace{q_{\rm R} n_{\rm R} + q_{\rm T} n_{\rm T}}_{\text{ion density}} = 1 \tag{24}$$

The resulting electric fields due to the presence of the ions further shift the energy levels by an amount

$$U_{\rm R(T)} n_{\rm ions} = U_{\rm R(T)}(q_{\rm R} n_{\rm R} + q_{\rm T} n_{\rm T}), \tag{25}$$

so that we obtain an overall rescaling of the detunings

$$\begin{aligned} D_{\rm R} \to D'_{\rm R} &= D_{\rm R} - \underbrace{(V_{\rm R} + q_{\rm R} U_{\rm R})}_{\equiv\alpha} n_{\rm R} - \underbrace{q_{\rm T} U_{\rm R}}_{\equiv\beta} n_{\rm T}, \\ D_{\rm RT} \to D'_{\rm RT} &= D_{\rm RT} - \underbrace{(V_{\rm T} + q_{\rm T} U_{\rm T})}_{\equiv\epsilon} n_{\rm T} - \underbrace{U_{\rm T} q_{\rm R}}_{\equiv\gamma} n_{\rm R}, \end{aligned} \tag{26}$$

where α, β, γ and ϵ are the four shift parameters introduced in the main text.

In order to find the stationary properties of this system we set all time derivatives to zero. Furthermore, in order to exploit the fact that the interactions considered here only amount to an effective energy shift, we proceed as follows: we first consider the non-interacting case $V_{\mathrm{R}} = V_{\mathrm{T}} = U_{\mathrm{R}} = U_{\mathrm{T}} = 0$ (corresponding to $\alpha = \beta = \gamma = \epsilon = 0$). This makes the equations linear: if we define a vector of expectations $\vec{v} = (n_{\mathrm{R}}, n_{\mathrm{T}}, \sigma^x_{\mathrm{R}}, \sigma^y_{\mathrm{R}}, \sigma^x_{\mathrm{RT}}, \sigma^y_{\mathrm{RT}}, \sigma^x_{\mathrm{T}}, \sigma^y_{\mathrm{T}})^{\intercal}$ then we can rewrite the non-interacting equations as $M\vec{v} = \vec{w}$, where

$$M = \begin{pmatrix} -\Gamma_{\mathrm{R}} & 0 & 0 & \Omega_{\mathrm{R}} & 0 & -\Omega_{\mathrm{T}} & 0 & 0 \\ 0 & -\Gamma_{\mathrm{T}} & 0 & 0 & 0 & \Omega_{\mathrm{T}} & 0 & 0 \\ 0 & 0 & -\frac{\Gamma_{\mathrm{R}}}{2} & D_{\mathrm{R}} & 0 & 0 & 0 & -\Omega_{\mathrm{T}} \\ -4\Omega_{\mathrm{R}} & -2\Omega_{\mathrm{R}} & -D_{\mathrm{R}} & -\frac{\Gamma_{\mathrm{R}}}{2} & 0 & 0 & \Omega_{\mathrm{T}} & 0 \\ 0 & 0 & 0 & 0 & -\frac{\Gamma_{\mathrm{R}}+\Gamma_{\mathrm{T}}}{2} & -D_{\mathrm{T}} & 0 & \Omega_{\mathrm{R}} \\ 2\Omega_{\mathrm{T}} & -2\Omega_{\mathrm{T}} & 0 & 0 & D_{\mathrm{T}} & -\frac{\Gamma_{\mathrm{R}}+\Gamma_{\mathrm{T}}}{2} & -\Omega_{\mathrm{R}} & 0 \\ 0 & 0 & 0 & -\Omega_{\mathrm{T}} & 0 & \Omega_{\mathrm{R}} & -\frac{\Gamma_{\mathrm{T}}}{2} & D_{\mathrm{RT}} \\ 0 & 0 & \Omega_{\mathrm{T}} & 0 & -\Omega_{\mathrm{R}} & 0 & -D_{\mathrm{RT}} & -\frac{\Gamma_{\mathrm{T}}}{2} \end{pmatrix} \tag{27}$$

and $\vec{w} = (0, 0, 0, -2\Omega_{\mathrm{R}}, 0, 0, 0, 0)^{\intercal}$. Now, if we define $M^{(i)}$ the matrix obtained by substituting $\vec{w}$ to the i-th column of M, one can get the expectation values by Cramer's rule; in particular, we call

$$n^{(0)}_{\mathrm{R}}(D_{\mathrm{R}}, D_{\mathrm{RT}}) = \frac{\det M^{(1)}}{\det M} \quad \text{and} \quad n^{(0)}_{\mathrm{T}}(D_{\mathrm{R}}, D_{\mathrm{RT}}) = \frac{\det M^{(2)}}{\det M}, \tag{28}$$

having highlighted the dependence on the detunings for later convenience. The expressions for these two quantities are rather long and will not be reported here. In order to understand their utility, let us consider a more general case: let

$$\vec{\mathcal{E}}(\vec{p}, \vec{v}) \equiv 0 \tag{29}$$

denote a set of N equations in the variables $\vec{v}$ (grouped in a N-component vector) and depending on some parameters $\vec{p}$ collected in a P-component vector. We shall denote a solution of this system as $\vec{v}^{(0)}_{\mathrm{sol}}(\vec{p})$. Let us also consider the equations obtained by shifting (possibly just some of) the parameters by amounts linear in the variables:

$$\vec{\mathcal{E}}(\vec{p} - A\vec{v}, \vec{v}) \equiv 0, \tag{30}$$

where A is some given $(P \times N)$ matrix. We now assume that, for some fixed parameters $\vec{p}$, this system admits a solution $\vec{v}_{\mathrm{sol}}(\vec{p})$, i.e.,

$$\mathcal{E}_i(\vec{p} - A\vec{v}_{\mathrm{sol}}(\vec{p}), \vec{v}_{\mathrm{sol}}(\vec{p})) = 0 \quad \forall\, i = 1 \ldots N. \tag{31}$$

If we now define $\vec{p}\,' = \vec{p} - A\vec{v}_{\rm sol}(\vec{p})$, we see that $\vec{v}_{\rm sol}(\vec{p})$ is a solution of the original set of supplementary equations (29) at parameters $\vec{p}\,'$, which implies that there exists a solution $\vec{v}^{(0)}_{\rm sol}$ such that

$$\vec{v}^{(0)}_{\rm sol}(\vec{p}\,') = \vec{v}_{\rm sol}(\vec{p}). \tag{32}$$

The converse is also true, since the supplementary set (29) can be obtained from the supplementary system (30) by just considering the inverse shift $\vec{p}\,' \to \vec{p} = \vec{p}\,' + A\vec{v}$. To apply this to our case, we associate our linear equations to the original system of supplementary equations (29) and our non-linear (supplementary) ones (16)-(23) to the second, shifted one (30). We can thus write

$$\begin{cases} n^{(0)}_{\rm R}(D'_{\rm R}, D'_{\rm RT}) = n_{\rm R}(D_{\rm R}, D_{\rm RT}) \\ n^{(0)}_{\rm T}(D'_{\rm R}, D'_{\rm RT}) = n_{\rm T}(D_{\rm R}, D_{\rm RT}), \end{cases} \tag{33}$$

with $D'_{\rm R}$ and $D'_{\rm RT}$ defined in Supplementary Equation (26). By fixing the shift parameters α, β, γ and ϵ these can now be interpreted as two equations for the determination of the two unknown variables $n_{\rm R}$ and $n_{\rm T}$

$$\begin{cases} n^{(0)}_{\rm R}(D_{\rm R} - \alpha n_{\rm R} - \beta n_{\rm T}, D_{\rm RT} - \gamma n_{\rm R} - \epsilon n_{\rm T}) = n_{\rm R} \\ n^{(0)}_{\rm T}(D_{\rm R} - \alpha n_{\rm R} - \beta n_{\rm T}, D_{\rm RT} - \gamma n_{\rm R} - \epsilon n_{\rm T}) = n_{\rm T} \end{cases} \tag{34}$$

and solved numerically. We remark that the solutions of the linear system $n^{(0)}_{\rm R(T)}$ do not depend linearly on their arguments $D'_{\rm R}$ and $D'_{\rm RT}$, which restores the original non-linearity of the problem, and thus permits the appearance of bifurcations and multiple solutions (bistability). The advantage is that it is numerically much simpler and less costly to look for solutions of the non-linear supplementary system (34) than it is to do so for the original supplementary one (16)-(23).
